# Modification of Threonine-825 of SlBRI1 Enlarges Cell Size to Enhance Fruit Yield by Regulating the Cooperation of BR-GA Signaling in Tomato

**DOI:** 10.3390/ijms22147673

**Published:** 2021-07-18

**Authors:** Shufen Wang, Siqi Lv, Tong Zhao, Meng Jiang, Dehai Liu, Shangtan Fu, Miaomiao Hu, Shuhua Huang, Yu Pei, Xiaofeng Wang

**Affiliations:** State Key Laboratory of Crop Stress Biology in Arid Areas, College of Horticulture, Northwest A&F University, Yangling, Xianyang 712100, China; shufenwang@nwafu.edu.cn (S.W.); lvsiqi163w@163.com (S.L.); 18829352848@163.com (T.Z.); jiangmeng412@163.com (M.J.); liudehai312000@163.com (D.L.); fyq2015678@163.com (S.F.); nwafu203044373@163.com (M.H.); hsh813@126.com (S.H.); peiyu7976@163.com (Y.P.)

**Keywords:** tomato, SlBRI1, Thr-825, fruit development, phosphorylation site

## Abstract

Brassinosteroids (BRs) are growth-promoting phytohormones that can efficiently function by exogenous application at micromolar concentrations or by endogenous fine-tuning of BR-related gene expression, thus, precisely controlling BR signal strength is a key factor in exploring the agricultural potential of BRs. BRASSINOSTEROID INSENSITIVE1 (BRI1), a BR receptor, is the rate-limiting enzyme in BR signal transduction, and the phosphorylation of each phosphorylation site of SlBRI1 has a distinct effect on BR signal strength and botanic characteristics. We recently demonstrated that modifying the phosphorylation sites of tomato SlBRI1 could improve the agronomic traits of tomato to different extents; however, the associated agronomic potential of SlBRI1 phosphorylation sites in tomato has not been fully exploited. In this research, the biological functions of the phosphorylation site threonine-825 (Thr-825) of SlBRI1 in tomato were investigated. Phenotypic analysis showed that, compared with a tomato line harboring SlBRI1, transgenic tomato lines expressing SlBRI1 with a nonphosphorylated Thr-825 (T825A) exhibited a larger plant size due to a larger cell size and higher yield, including a greater plant height, thicker stems, longer internodal lengths, greater plant expansion, a heavier fruit weight, and larger fruits. Molecular analyses further indicated that the autophosphorylation level of SlBRI1, BR signaling, and gibberellic acid (GA) signaling were elevated when SlBRI1 was dephosphorylated at Thr-825. Taken together, the results demonstrated that dephosphorylation of Thr-825 can enhance the functions of SlBRI1 in BR signaling, which subsequently activates and cooperates with GA signaling to stimulate cell elongation and then leads to larger plants and higher yields per plant. These results also highlight the agricultural potential of SlBRI1 phosphorylation sites for breeding high-yielding tomato varieties through precise control of BR signaling.

## 1. Introduction

Tomato (*Solanum lycopersicum*) is one of the most important vegetables worldwide, and fruit yield, including fruit number and fruit weight, is the major agronomic trait of concern for tomato breeding. Numerous studies have shown that whole tomato fruit growth is essentially based on cell division and expansion, which rely on multiple hormonal signals and relevant responsive genes [1,2,3]. For example, the cell division stage is tightly controlled by cyclin-dependent kinases (CDKs) [4,5]. Plant hormones such as auxin (indole-3-acetic acid, IAA), gibberellic acids (GAs), and brassinosteroids (BRs) have been repeatedly demonstrated to be major regulators of cell division and expansion [6,7,8]. Exogenous application of these hormones and endogenous expression of their regulatory genes can alter both fruit growth and development in tomato [9,10,11].

BRs, plant steroid hormones, regulate cell division, differentiation, and expansion, thereby influencing seed formation and germination, root development, lateral shoot formation, floral organ development, and so on [12,13]. BRI1 is a leucine-rich repeat receptor-like kinase (LRR-RLK) located on the plasma membrane and acts as the rate-limiting receptor in BR signaling [14,15]. In the presence of BRs, which are first perceived by BRI1, phosphorylation cascades of the signal components can activate the critical transcription factors BRASSINAZOLERESISTANT1 (BZR1) and BRI1-EMS SUPPRESSOR1 (BES1), which bind to target promoters of BR-responsive genes to modulate BR signaling and the consequent BR-regulated biological processes [16,17,18]. Numerous studies in different species have reported that BRI1 homolog-deficient mutants exhibit BR signaling inhibition and common characteristics to various degrees, including dwarfism, vertical growth, dark green leaves, and male sterility [19,20,21,22].

During BR signal transduction, BRI1 is a dual-specificity kinase capable of phosphorylating both serine/threonine and tyrosine residues; thus, phosphorylation sites in BRI1 are important for the biological function of BRI1. Most phosphorylation sites play different roles by regulating the kinase activities of BRI1 and BR signal strength. For example, Thr-1039 and Ser-1044 had a vital role in regulating kinase activation, and the *Arabidopsis thaliana* BRI1 weak mutant *bri1-5* transformed with dephosphorylated Thr-1039 of BRI1 (T1039A, in which Thr-1039 was replaced with Ala) and S1044A showed aborted BR signaling and kinase activity, which resulted in aggravated dwarfism and sterility of plants. In contrast, Tyr-831, Ser-1172, and Ser-1187 showed more subtle effects on the kinase activities of BRI1 and BR signal strength, and dephosphorylation of these sites in *Arabidopsis* resulted in enhanced vegetative growth, which has potential for Chinese cabbage breeding [23,24,25]. Notably, *bri1-5* expressing S1042A, with partly inhibited kinase activity of BRI1 and BR signaling, showed a semidwarf phenotype and reduced plant expansion but increased seed yield per plant compared with the wild-type, indicating the potential in dwarf breeding [25]. Compared with the findings on the above sites, mutation of the phosphorylation sites with a weaker influence on the kinase activation of BRI1 and BR signaling moderately affected plant growth and therefore might have much more potential to improve agronomic traits.

Tomato is a model berry crop, and tomato SlBRI1 is important for BR signaling and plant growth and development. The SlBRI1 weak mutant *cu3^-abs1^* showed inhibited BR signaling, as well as a semidwarf phenotype, a dark green color, winkled leaves, and weak fertility. Overexpressing SlBRI1 in tomato could improve fruit yield and nutritional quality [26,27]. Further studies suggested that most phosphorylation sites in SlBRI1 were conserved with those in *Arabidopsis* BRI1, and the functions of partially conserved phosphorylation sites in SlBRI1, such as Thr-1054 (equivalent to *Arabidopsis* BRI1 Thr-1049), Thr-1050 (equivalent to *Arabidopsis* BRI1 Thr-1045), and Ser-1040 (equivalent to *Arabidopsis* BRI1 Ser-1035) were investigated. Dephosphorylation of SlBRI1 Thr-1054 (T1054A), T1050A, and S1040A completely abolished, enhanced, and partly inhibited the kinase activity of SlBRI1, respectively. These sites have distinguishing functions in tomato growth. Transgenic *cu3^-abs1^* tomato expressing T1054A did not show rescue of the *cu3^-abs1^* phenotype and displayed even more severe dwarfism and male sterility [26,28]. *cu3^-abs1^* expressing T1050A showed enhanced plant expansion, leaf area, and fruit yield but decreased tomato nutrients compared with *cu3^-abs1^* expressing wild-type SlBRI1 [29]. *cu3^-abs1^* expressing S1040A showed a similar phenotype but improved yield and tolerance under high temperature, compared with *cu3^-abs1^* with wild-type SlBRI1 [30]. In conclusion, phosphorylation sites of SlBRI1 play key roles in the function of SlBRI1, which are different from those in *Arabidopsis* BRI1, and each site functions distinctly during plant growth and development and plays different roles by diverse pathways. However, functional studies of the phosphorylation sites of SlBRI1 in tomato are still limited, and the roles of the phosphorylation sites of SlBRI1 in regulating the cooperation of BR-GA signaling remain unknown.

In this research, the biological function of the Thr-825 phosphorylation site in SlBRI1 in tomato was investigated. The Thr-825 residue is a unique phosphorylation site of SlBRI1 since no corresponding residue has been identified in AtBRI1. Autophosphorylation analysis showed that Thr-825 played a negative role in the phosphorylation of SlBRI1 and BR signaling. Transgenic tomato lines expressing T825A in the *cu3^-abs1^* mutant background showed enhanced plant growth and tomato yields through effects on single fruit development, as well as enhanced GA signaling. These results suggested the unique biological function of Thr-825. Dephosphorylation of Thr-825 could enhance the autophosphorylation level of SlBRI1 and BR signal strength and then elevate GA signaling and the expression of cell cycle-regulated genes. These factors coordinately promote cell division and expansion and eventually led to the larger plant shape and increased fruit yield of tomato. Finally, these results are important not only for systematically identifying the function of SlBRI1 phosphorylation sites in tomato, but also for revealing the regulatory mechanism of SlBRI1 in cooperation with BR-GA signaling and plant growth.

## 2. Results

### 2.1. SlBRI1 Thr-825 Influences Autophosphorylation of SlBRI1

**Figure 1:**
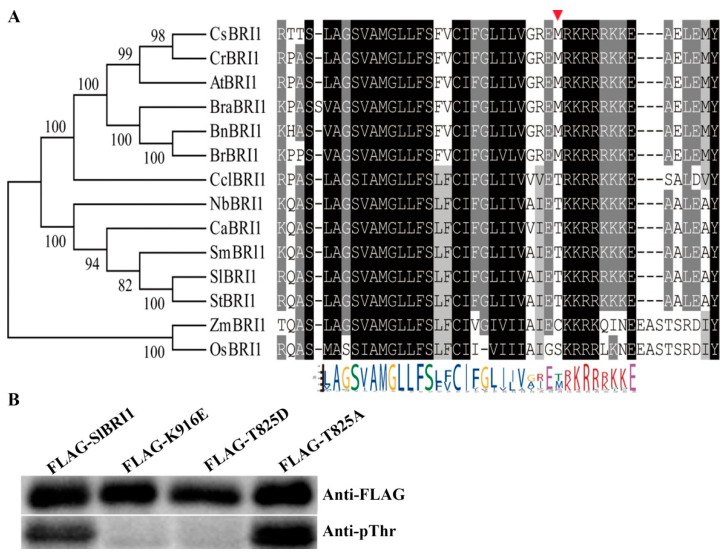
SlBRI1 Thr-825 influences the autophosphorylation of SlBRI1. (**A**) Sequence alignments of the partial kinase domains of BRI1 homologs. Gray and black regions indicate similar and conserved amino acids among BRI1 homologs, respectively. The red triangle shows the series of Thr-825 amino acids on BRI1 homologs. Conserved amino acids at individual sites are distinguished by different symbols with colors on the bottom. AtBRI1 (*Arabidopsis thaliana*, NP_195650.1), SlBRI1 (*Solanum lycopersicum*, NP_001296180.1), StBRI1 (*Solanum tuberosum*, XP_006357355.1), SmBRI1 (*Solanum melongena L.,* Sme2.5_00067.1_g00001.1), NtBRI1 (*Nicotiana tabacum,* NP_001312072.1), BrBRI1 (*Brassica rapa,* XP_009101880.2), BraBRI1 (*Brassica oleracea var. oleracea*, XP_013597742.1), BnBRI1 (*Brassica napus*, NP_001303105.1), OsBRI1 (*Oryza sativa*, NP_001044077.1), ZmBRI1 (*Zea mays*, XP_008656807.1), CsBRI1 (*Camelina sativa*, XP_010431911.1), CrBRI1 (*Capsella rubella,* XP_006282536.1), CclBRI1 (*Citrus clementina*, XP_006427932.1), and CaBRI1 (*Capsicum annuum*, KAF3634706.1). (**B**) Autophosphorylation level of SlBRI1 in vitro. Western blotting analysis of the autophosphorylation levels of the recombinant FLAG-SlBRI1, FLAG-K916E, FLAG-T825D, and FLAG-T825A proteins was detected using anti-pThr antibodies, as shown on the bottom. The protein loading levels were detected using anti-FLAG antibodies, as shown on the top.

SlBRI1 is a receptor kinase that exerts its biological function through phosphorylation, and its phosphorylation sites have different effects on the phosphorylation level and mediate the biological functions of SlBRI1 [29,30]. Thus, conservative analysis of SlBRI1 Thr-825 was first performed to preliminarily assess its function in tomato. Amino acid sequence analysis of SlBRI1 homologs showed that Thr-825 was conserved in tobacco, eggplant, pepper, and potato, which belong to the Solanaceae family of plants, indicating that Thr-825 might play a special biological function in tomato (Figure 1A).

We further assessed the importance of Thr-825 for the biological functions of SlBRI1. Autophosphorylation levels of SlBRI1, a kinase-inactive form of SlBRI1 (K916E), T825A, and T825D in vitro were compared. The intensities of the phosphorylation bands from strong to weak were as follows: T825A, SlBRI1, T825D, and K916E (Figure 1B). This result suggested that Thr-825 has a negative effect on SlBRI1 autophosphorylation.

### 2.2. SlBRI1 T825A Affects BR Signaling

**Figure 2:**
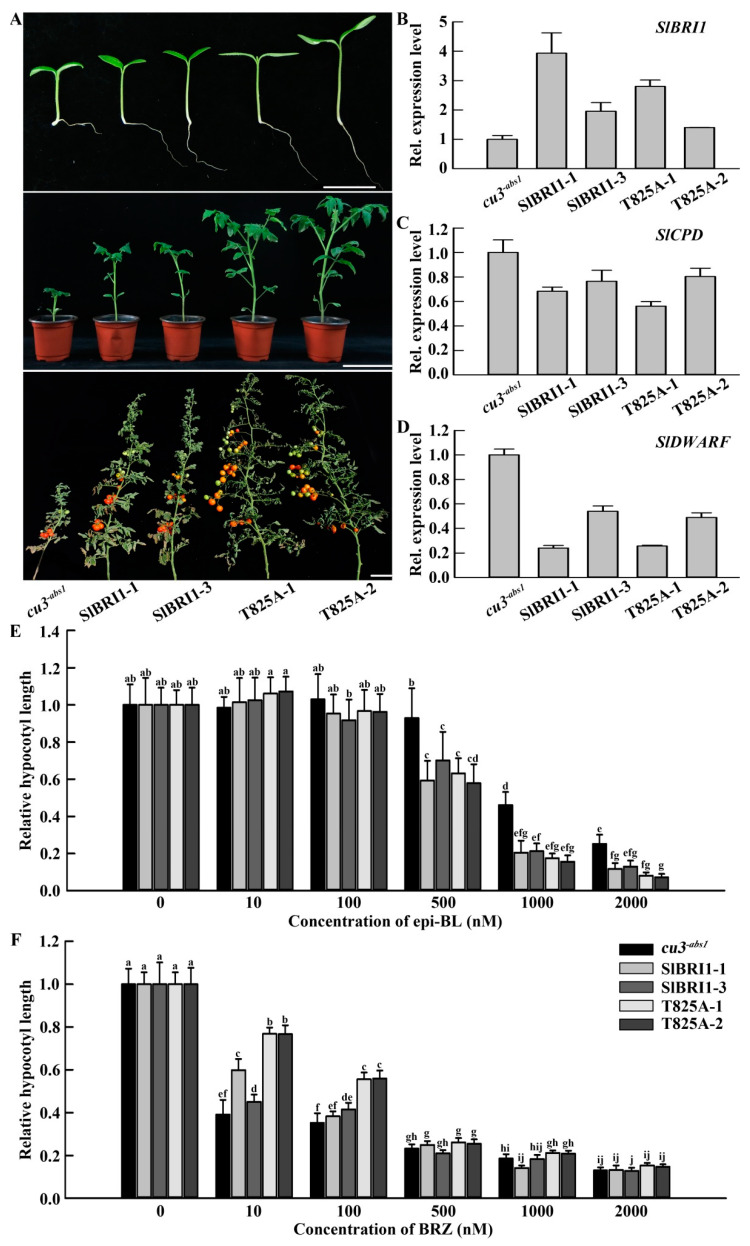
SlBRI1 T825A affects BR signaling. (**A**) The plant phenotypes. From top to bottom: phenotypes of plants at the germination stage (7 days after sowing), seedling stage (42 days after sowing), and maturation stage (maturation period of the fourth fruit cluster of the P*_SlBRI1_*::T825A lines). Scale bars represent 2 cm, 10 cm, and 14 cm, respectively. (**B**) Relative transcript levels of *SlBRI1* were tested by qRT-PCR. (**C**,**D**) Relative transcript levels of *SlCPD* (**C**) and *SlDWARF* (**D**) as BR signaling marker genes were tested by qRT-PCR. *SlACTIN* was used as a reference gene in tomato. (**E**,**F**) Dose-response levels of relative hypocotyl lengths of 10-day-old tomato seedlings treated with increasing concentrations of exogenous epi-BL (**E**) and BRZ (**F**). Tomato seedlings grew on solid 1/2 strength MS medium in the dark at 25 °C. Each data point for (**B**–**D**) had 3 biological and technical replications. The data for (**E**,**F**) are presented as the mean ± SD (*n* = 15). The different letters indicate significant differences (one-way ANOVA, Tukey’s test, *p* < 0.05).

SlBRI1 is a BR receptor that initiates BR signaling and functions in plant growth through its phosphorylation sites. To determine the biological function of Thr-825 in tomato, transgenic tomato lines expressing SlBRI1 (SlBRI1-1 and SlBRI1-3) or T825A (T825A-1 and T825A-2) were generated. For phenotypic analysis, P*_SlBRI1_*::SlBRI1 and *cu3^-abs1^* were considered the positive control and negative controls of P*_SlBRI1_*::T825A, respectively. Phenotypic observation showed that dephosphorylation of both Thr-825 and SlBRI1 could rescue the semidwarf phenotype of *cu3^-abs1^* (Figure 2A). The transcript levels of SlBRI1 in transgenic lines were all higher than those in *cu3^-abs1^*; furthermore, the transgenic lines SlBRI1-1 and T825A-1, as well as SlBRI1-3 and T825A-2, presented similar SlBRI1 expression levels (Figure 2B).

To determine whether Thr-825 affects BR signaling in tomato, the expression levels of the BR biosynthetic genes *SlCPD* and *SlDWARF* were assessed, together with the BR sensitivity of seedlings, to compare BR signal intensities. The results showed that transcription of *SlCPD* and *SlDWARF*, which were feedback-inhibited by BR signals, was similar in the transgenic lines but lower than that in *cu3^-abs1^* (Figure 2C,D). In addition, the relative hypocotyl lengths under six increasing concentrations of epi-BL or BRZ were compared to quantitatively evaluate the BR signal intensities among the tomato plants. As shown in Figure 2E, *cu3^-abs1^* was insensitive to epi-BL since its hypocotyl lengths were nearly unchanged when the concentration of epi-BL was below 500 nM, but the lengths decreased by 53.9% and 74.7% with concentrations of 1000 nM and 2000 nM, respectively. The P*_SlBRI1_*::SlBRI1 and P*_SlBRI1_*::T825A seedlings showed the same trend of change under the different concentrations of epi-BL; the hypocotyl lengths were increased by 2.0% and 6.6% when the concentration was 10 nM, respectively, while they decreased by 6.4% and 3.4%, 35.2% and 39.4%, 79.1% and 83.4%, and 87.6% and 92.3% with concentrations of 100 nM, 500 nM, 1000 nM, and 2000 nM, respectively (Figure 2E). The results of the hypocotyl elongation response to BRZ treatment were consistent with those to epi-BL; the hypocotyl lengths of *cu3^-abs1^* showed the greatest decreases, while those of P*_SlBRI1_*::SlBRI1 and P*_SlBRI1_*::T825A were decreased by 47.6% and 23.3%, 60.1% and 44.3%, 77.0% and 74.2%, 83.7% and 79.0%, and 86.9% and 84.9% with concentrations of 10 nM, 100 nM, 500 nM, 1000 nM, and 2000 nM, respectively (Figure 2F).

### 2.3. SlBRI1 T825A Promotes Plant Growth

**Figure 3:**
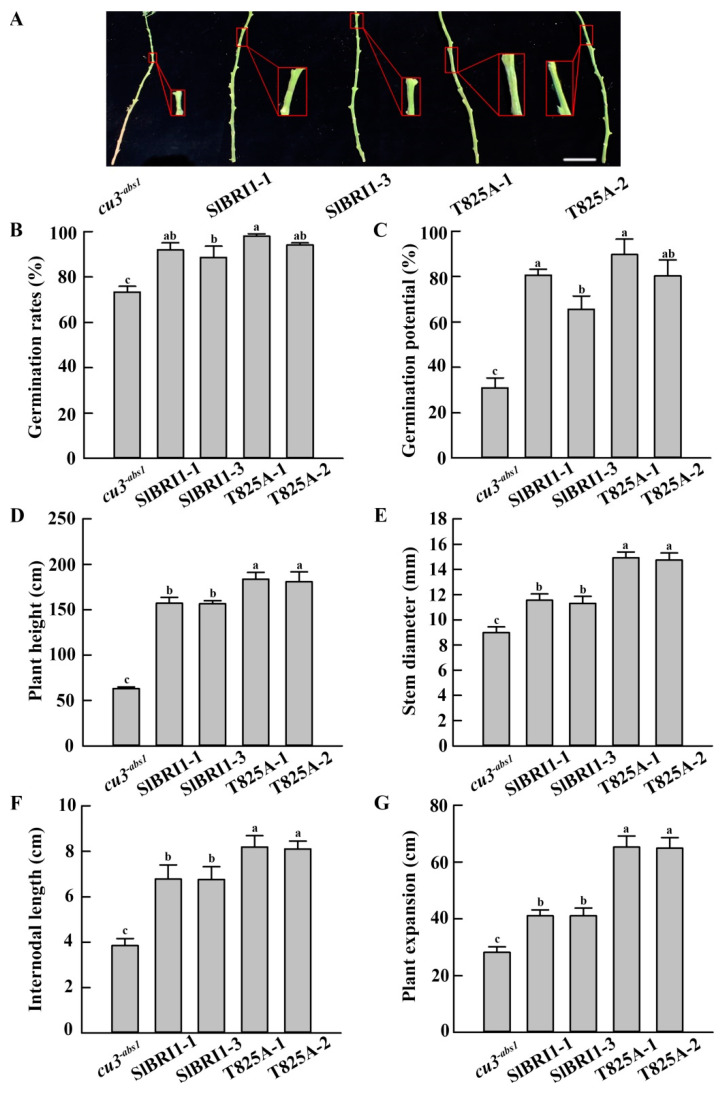
SlBRI1 T825A promotes plant growth. (**A**) Phenotypes of plant stems at the mature stage. The maturation period of the fourth fruit cluster of the P*_SlBRI1_*::T825A lines was regarded as the mature period. The red rectangular box indicates the eleventh fruit nodes from bottom to top per plant under a 2.5x-magnified visual field. Scale bar represents 14 cm. (**B**,**C**) Germination rates (**B**) and the germination potential (**C**) of plants on the seventh day and third day after sowing at 28 °C in the dark, respectively. (**D**–**G**) Plant height (**D**), stem diameter (**E**), internodal length (**F**), and plant expansion (**G**) per plant at the mature stage. The data for (**B**,**C**) are presented as the mean ± SD (*n* = 3), and each replicate had 100 seeds. The data for (**D**,**E**) are presented as the mean ± SD (*n* = 10). The data for (**F**,**G**) are presented as the mean ± SD (*n* = 15). The different letters indicate significant differences (one-way ANOVA, Tukey’s test, *p* < 0.05).

Agronomic traits, including seed germination, plant height, stem diameter, internodal length, and plant expansion were further analyzed to evaluate the influence of Thr-825 on the functions of SlBRI1 during plant growth. The germination rates and the germination potential of the P*_SlBRI1_*::T825A lines were 6% and 16% higher than those of P*_SlBRI1_*::SlBRI1 lines, respectively (Figure 3B,C). The plant heights and stem diameters of the P*_SlBRI1_*::T825A lines were 1.16- and 1.30-fold higher than those of the P*_SlBRI1_*::SlBRI1 lines at the mature stage, respectively (Figure 3D,E). The internodal length and plant expansion of P*_SlBRI1_*::T825A were 1.20- and 1.58-fold those of P*_SlBRI1_*::SlBRI1 at the mature stage, respectively (Figure 3F,G). Taken together, these results suggest that dephosphorylation of Thr-825 can promote the functions of SlBRI1 in plant growth.

### 2.4. SlBRI1 T825A Improves Tomato Yields

**Figure 4:**
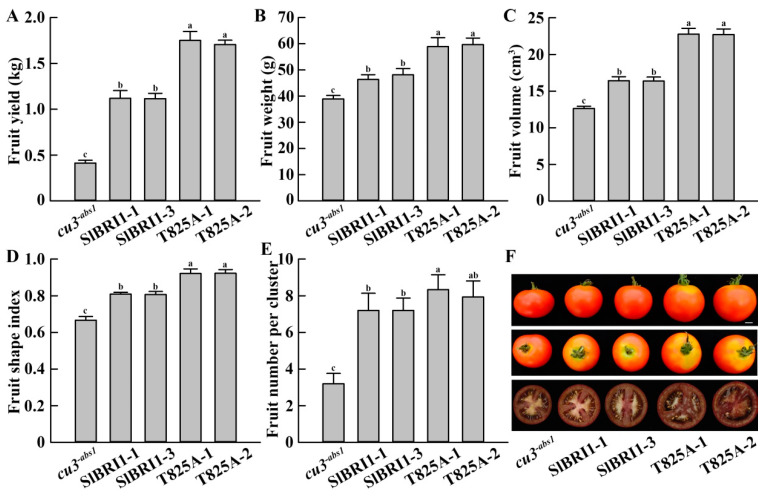
SlBRI1 T825A improves tomato yields. (**A**) Fruit yield per plant for the first to fourth fruit nodes. (**B**–**D**) Single fruit weight (**B**), fruit volume (**C**), and fruit shape index (**D**) at the red ripening stage. The fruit shape index is the ratio of the longitudinal diameter to the transverse diameter of the fruit. (**E**) Fruit number per cluster for the third cluster. (**F**) Top, phenotypes of single fruits with a front view. Middle, phenotypes of single fruits with a top view. Bottom, the equatorial sections of single fruits. Scale bar represents 1 cm. The data for (**A)** are presented as the mean ± SD (*n* = 6). The data for (**B**–**E**) are presented as the mean ± SD (*n* = 15). The different letters indicate significant differences (one-way ANOVA, Tukey’s test, *p* < 0.05).

To determine the effects of Thr-825 on tomato yield, fruit yield per plant was investigated. The results showed that the P*_SlBRI1_*::T825A plants exhibited 1.55- and 4.18-fold higher fruit yields per plant than the P*_SlBRI1_*::SlBRI1 and *cu3^-abs1^* plants, respectively (Figure 4A). The correlating traits, including fruit weight, fruit volume, fruit shape index, and fruit number per cluster, were subsequently analyzed to reveal the factors leading to the differences in fruit yield. The single fruit weight for P*_SlBRI1_*::T825A was 18% and 36% heavier than that for P*_SlBRI1_*::SlBRI1 and *cu3^-abs1^* because the fruit volume of the former was 28% and 45% larger, respectively (Figure 4B,C). In addition, the fruit shape index of P*_SlBRI1_*::T825A was increased by 13% and 28%, and the fruit number per cluster also increased by 10% and 61%, respectively, compared with those of P*_SlBRI1_*::SlBRI1 and *cu3^-abs1^* (Figure 4D–F). Moreover, the fruits of P*_SlBRI1_*::T825A showed the thickest pericarp among the observed lines (Figure 4F). Overall, these results suggested that the increased yield of P*_SlBRI1_*::T825A was due mainly to the different fruit weights.

### 2.5. SlBRI1 T825A Influences Early Fruit Development

**Figure 5:**
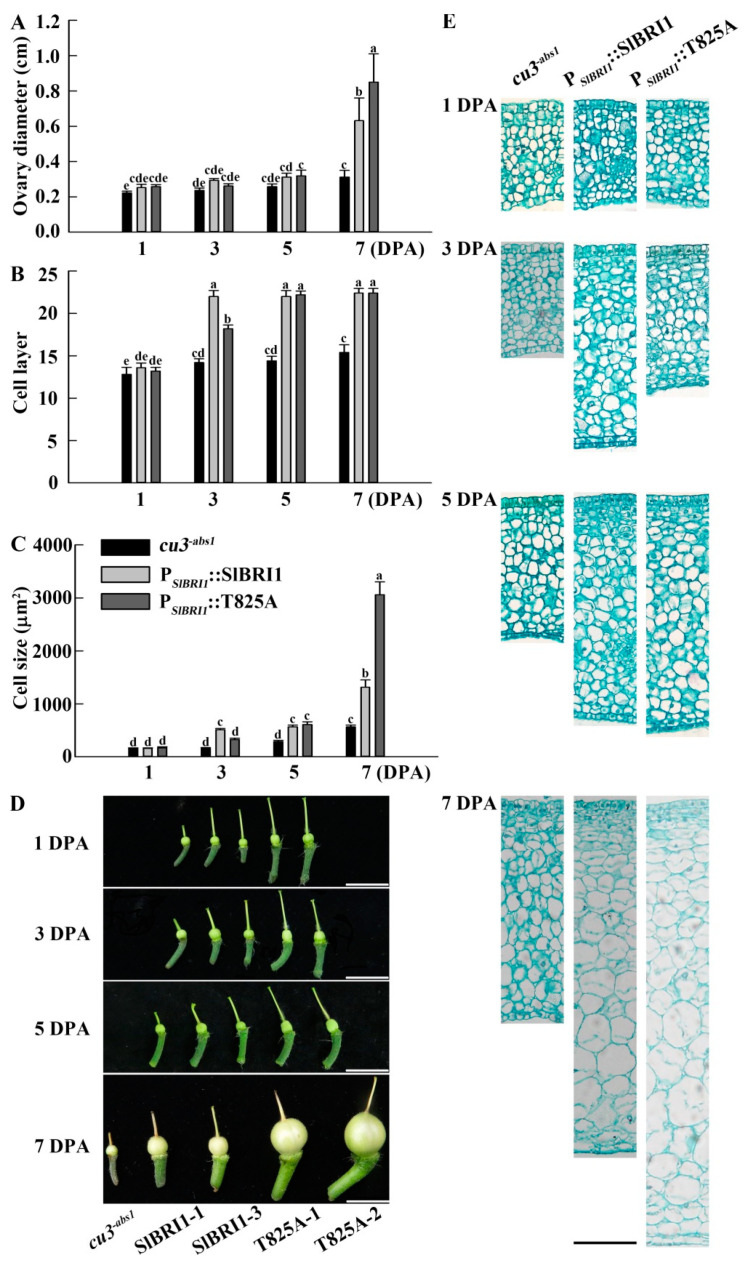
SlBRI1 T825A influences early fruit development. (**A**) Ovary diameters per fruit at 1 DPA, 3 DPA, 5 DPA, and 7 DPA. (**B**,**C**) The cell layer (**B**) and cell size (**C**) of equatorial sections of the ovary walls per fruit at 1 DPA, 3 DPA, 5 DPA, and 7 DPA. (**D**) Phenotypes of ovary per fruit at 1 DPA, 3 DPA, 5 DPA, and 7 DPA. Scale bars represent 1 cm. (**E**) Phenotypes of the equatorial sections of the ovary walls per fruit at 1 DPA, 3 DPA, 5 DPA, and 7 DPA. Scale bar represents 100 μm. The data for (**A**) are presented as the mean ± SD (*n* = 15). The data for (**B**,**C**) are presented as the mean ± SD (*n* = 5). The different letters indicate significant differences (one-way ANOVA, Tukey’s test, *p* < 0.05).

To determine how Thr-825 phosphorylation extensively affects fruit weight, the ovary wall at 1 DPA, 3 DPA, 5 DPA, and 7 DPA was analyzed. The results showed that ovary diameter, ovary wall cell layer, and ovary wall cell size were similar among the observed lines at 1 DPA (Figure 5). The ovary wall cells of P*_SlBRI1_*::SlBRI1 divided and expanded most quickly, since the cell layer and cell size at 3 DPA were 18% and 35% and 36% and 68% higher than those of P*_SlBRI1_*::T825A and *cu3^-abs1^*, respectively (Figure 5B,C,E). No significant differences in ovaries were observed among the transgenic lines at 5 DPA, while *cu3^-abs1^* showed a slower speed of cell division and expansion (Figure 5). At 7 DPA, the ovary diameter of P*_SlBRI1_*::T825A was 1.34- and 2.72-fold that of P*_SlBRI1_*::SlBRI1 and *cu3^-abs1^*, respectively, and these differences were mostly due to the 2.32-fold and 5.41-fold larger cell sizes (Figure 5A,C). The cell layer for P*_SlBRI1_*::T825A was similar to that of P*_SlBRI1_*::SlBRI1 and only 31% higher than that of *cu3^-abs1^* at 7 DPA (Figure 5B).

### 2.6. SlBRI1 T825A Affects Expression of Fruit Growth-Related Genes

**Figure 6:**
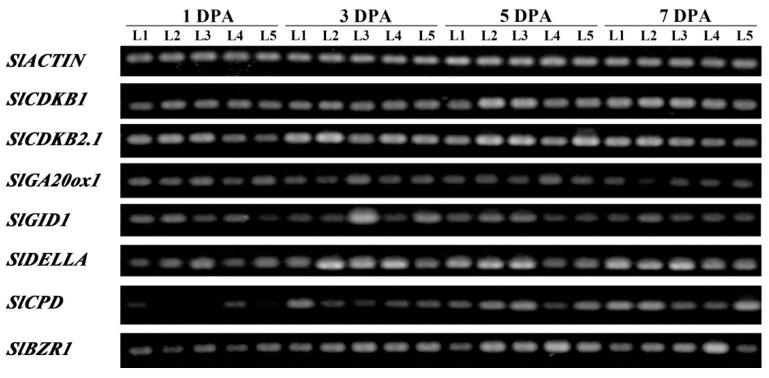
SlBRI1 T825A affects the expression of fruit growth-related genes. The relative transcript levels of fruit growth-related genes in ovaries at 1 DPA, 3 DPA, 5 DPA, and 7 DPA from both the transgenic and *cu3^-abs1^* plants were tested by RT-PCR. *SlACTIN* was used as a reference gene in tomato. L1 (Line 1), *cu3^-abs1^*; L2 (Line 2), SlBRI1-1; L3 (Line 3), SlBRI1-3; L4 (Line 4), T825A-1; L5 (Line 5), T825A-2. Each product had 3 biological replications.

Fruit development consists of a series of physiological and biochemical changes that involve multiple signaling pathways and large numbers of genes [1,2,3]. To determine the regulatory pathway of Thr-825 in fruit development, the expression levels of fruit growth-related genes in ovaries at 1 DPA, 3 DPA, 5 DPA, and 7 DPA by RT-PCR were analyzed. Characterization of the expression of CDKs that affects fruit growth through cell division regulation indicated that *SlCDKB2.1* was repressed in the ovaries of P*_SlBRI1_*::T825A compared with those of P*_SlBRI1_*::SlBRI1, while *SlCDKB1* mRNA accumulation was not altered. Furthermore, the expression of the GA-biosynthetic gene *SlGA20ox1* was similar, while the GA receptor gene *SlGID1* (*GIBBERELLIN INSENSITIVE DWARF 1*) and the GA signaling repressor gene *SlDELLA* was repressed in the ovaries of P*_SlBRI1_*::T825A after 5 DPA compared with those in the P*_SlBRI1_*::SlBRI1 ovaries. In addition, compared with that in the ovaries of P*_SlBRI1_*::T825A, the expression of the BR biosynthetic gene *SlCPD* in the P*_SlBRI1_*::SlBRI1 ovaries was repressed at 3 DPA and induced at 5 DPA, while the expression of the BR-regulated transcription factor gene *SlBZR1* was similar at 3 DPA and slightly induced after 3 DPA (Figure 6).

### 2.7. SlBRI1 T825A, S1040A, and T1050A Differentially Affect BR and GA Signaling

**Figure 7:**
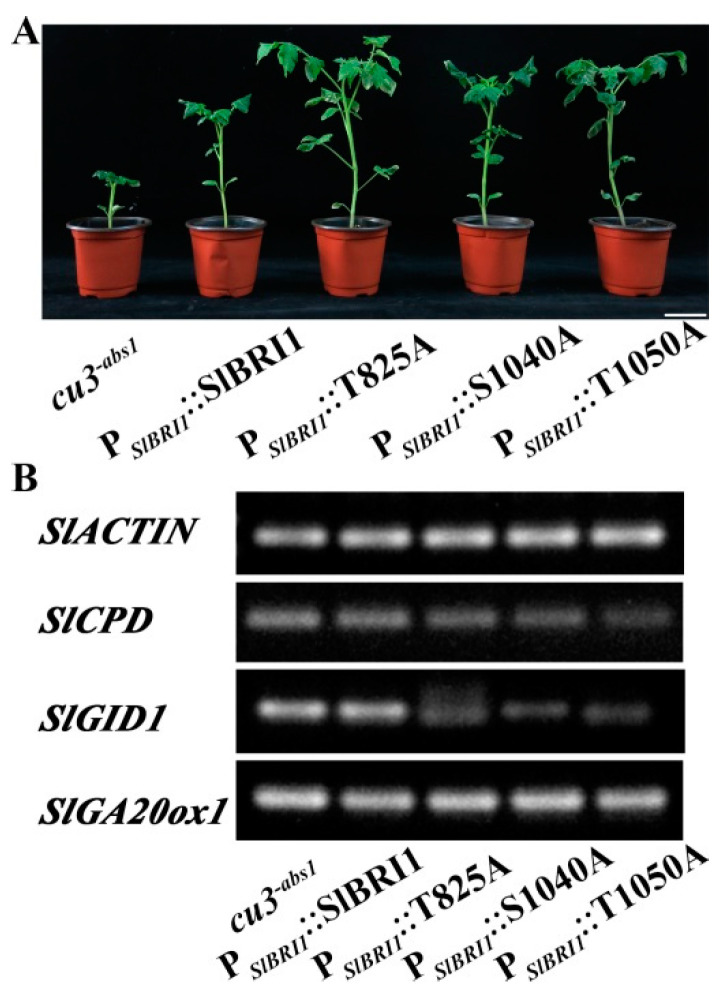
Comparison of the effects of SlBRI1 T825A, S1040A, and T1050A on BR and GA signaling. (**A**) Phenotypes of plants at the seedling stage (42 days after sowing), Scale bar represents 5 cm. (**B**) Relative transcript levels of *SlCPD*, *SlGID1*, and *SlGA20ox1* in the second leaves from both the transgenic and *cu3^-abs1^* plants at the six-leaf stage were tested by RT-PCR. *SlACTIN* was used as a reference gene in tomato. Each product had 3 biological replications.

To determine whether only Thr-825 functioned through the correlation of BR and GA signaling, the expression levels of BR-related genes and GA-related genes were compared in the leaves of the P*_SlBRI1_*::T825A, P*_SlBRI1_*::S1040A, P*_SlBRI1_*::T1050A, and P*_SlBRI1_*::SlBRI1 transgenic and *cu3^-abs1^* plants. For GA signaling-related genes, the expression of *SlGID1* was found to be decreased in the P*_SlBRI1_*::T825A plants, P*_SlBRI1_*::S1040A and P*_SlBRI1_*::T1050A plants compared with those in the P*_SlBRI1_*::SlBRI1 plants. For BR signaling-related genes, the expression of *SlCPD* shown from high to low is as follows: P*_SlBRI1_*::SlBRI1, P*_SlBRI1_*::T825A, P*_SlBRI1_*::S1040A, and P*_SlBRI1_*::T1050A (Figure 7B). These results suggested that Thr-825, Ser-1040, and Thr-1050 differentially affect GA signaling, and the effect of SlBRI1 Thr-825 was most obvious.

## 3. Discussion

Tomatoes are indispensable for the human diet, and high yield is considered one of the major breeding objectives of tomato varieties [31]. Previous studies of different species suggest that BRI1 homologs affect the per unit yield in two different ways: either increasing individual plant yield or maintaining the individual plant yield but allowing an increased planting density. For the first strategy, previous studies showed that overexpression of BRI1 in *Arabidopsis* could promote plant growth and consequently increase the seed yield per plant, as could SlBRI1 in tomato [25,27]. For the second strategy, previous studies showed that the weak OsBRI1 mutant in rice, ZmBRI1-RNAi transgenic maize, and the weak HvBRI1 mutant in barley displayed a semidwarf and compact stature, combined with slightly reduced fertility, which was beneficial for dense planting conditions and consequently increased the per unit yield [21,32,33,34].

This research focused on the effects of SlBRI1 phosphorylation sites on the function of SlBRI1. Each phosphorylation site influenced the function of SlBRI1 in different ways and mechanisms, and they are all linked together and coordinate with each other to mediate the biological functions of SlBRI1 in tomato. Previous studies in *Arabidopsis* have shown that phosphorylation sites of BRI1 could influence the yield-increasing function of BRI1 to different degrees, with individual plant yields of transgenic plants shown from high to low as follows: S1042A, BRI1, T1039A, S858A, S1187A, T872A, S887A, T842A, S1168A, S1044A, and S1172A [25]. Recent studies in tomato demonstrated that phosphorylation sites of SlBRI1 influence the yield mainly by regulating the fruit number per cluster. For example, compared with the *cu3^-abs1^* plants harboring SlBRI1, the *cu3^-abs1^* plants expressing dephosphorylated Thr-1054 showed a severe dwarf phenotype and sterility, while the *cu3^-abs1^* plants expressing Thr-1050-Ala and Ser-1040-Ala exhibited higher fruit yield mainly through more fruits per cluster [26,29,30]. In this study, the individual plant yield of the P*_SlBRI1_*::T825A lines was 1.6-fold that of the P*_SlBRI1_*::SlBRI1 lines, and the increased yield was mostly due to the effects in a single fruit. Since the fruit weight and fruit volume increased by 18% and 28%, respectively, both parameters showed greater changes than the fruit number per cluster, which was 10% (Figure 4). These results suggested that Thr-825 affected fruit yield through fruit growth more than fruit setting, in contrast to Thr-1050 and Ser-1040.

After fertilization, the early tomato fruit developmental stage can be divided into the division and expansion processes. Cell division of the ovary wall, which forms the pericarp, starts at 2 DPA, while the cell layer is determined at approximately 3–6 DPA, with the cell expansion period beginning subsequently [2,35]. Both stages are critical for tomato fruit formation, since the fruit size and weight are mainly dependent on the cell number and size of the pericarp, which are determined by cell division and expansion. In this study, the fruits of the P*_SlBRI1_*::T825A lines were heavier and larger than those from the P*_SlBRI1_*::SlBRI1 lines, and analysis of the paraffin sections of the ovaries further indicated that this difference was due to cell expansion more than cell division. The ovary wall cells from P*_SlBRI1_*::T825A were 2.32-fold larger than those from P*_SlBRI1_*::SlBRI1, while their cell layers were similar (Figure 5). These results suggested that Thr-825 influenced fruit development through regulation of cell expansion. Moreover, the stem diameter and internodal length of P*_SlBRI1_*::T825A were 1.3- and 1.2-fold greater than those of P*_SlBRI1_*::SlBRI1; these differences mostly resulted from the larger cell size, as the cell length and cross-section area of the stems in P*_SlBRI1_*::T825A were 1.24- and 1.15-fold those in P*_SlBRI1_*::SlBRI1 (Figure S1). Furthermore, the sepals, petals, stamens, and pistils of the P*_SlBRI1_*::T825A lines among the floral organs were larger than those of the P*_SlBRI1_*::SlBRI1 lines at different levels (Figure S2). Taken together, these results demonstrated that dephosphorylation of Thr-825 can promote cell expansion and that this promotion is conserved between vegetative growth and fruit development.

Previous studies have demonstrated that the cell division stage of fruit development is tightly controlled by the cell cycle machinery, and BR is believed to promote cell elongation by enhancing the expression of cell cycle regulatory genes. The expression of CDKs, which are key regulators of cell development, was upregulated by BRs in the dark but remained unchanged in the light [4,5,36]. In tomato fruits, *CDKB* genes were highly expressed before 15 DPA and related to cell division during this stage [37,38]. However, either overexpression or loss-of-function of *CDKBs* resulted in a similar reduction in cell division, probably due to the disruption of hormone pathways or interactions of CDK and its cyclin proteins, inhibiting proper cell cycle progression [39,40]. In this study, *SlCDKB2.1* was repressed in the ovaries of P*_SlBRI1_*::T825A compared with those of P*_SlBRI1_*::SlBRI1; however, these expression differences were slight and narrowed during fruit growth (Figure 6). This result could well explain why the ovary wall cells of P*_SlBRI1_*::SlBRI1 divided most quickly before 5 DPA but were similar to those of P*_SlBRI1_*::T825A at 7 DPA (Figure 5). Taken together, the results indicated that Thr-825 could affect cell division by regulating the expression of *SlCDKB2.1* at a very early stage of fruit development.

BRs are found at relatively high concentrations in young organs and function at nanomolar concentrations in cell division and elongation [41,42,43,44]. BR-insensitive mutants of different species all showed inhibited cell elongation, so with a dwarf phenotype, BR signaling could significantly promote callus volume in rice, as well as balance cell division and expansion in the leaf of *Arabidopsis* [13,45]. In this study, the larger fruit of the P*_SlBRI1_*::T825A lines was mainly due to their stronger BR signaling for the following reasons. First, the P*_SlBRI1_*::T825A plants were more sensitive to epi-BR and insensitive to BRZ, since their relative changes in hypocotyl length were the largest after treatment with epi-BR and the smallest under BRZ treatment among all the lines, which was consistent with previous studies [46]. Second, the transcription levels of *SlCPD* and *SlDWARF*, which are feedback-inhibited by BR signals [47,48], were similar in the transgenic lines but lower than those in *cu3^-abs1^* (Figure 2C,D). In addition, the transcription level of *SlCPD* in the P*_SlBRI1_*::SlBRI1 ovaries was repressed at 3 DPA and induced at 5 DPA compared to that in the P*_SlBRI1_*::T825A ovaries (Figure 6). This difference could correspond to the greater cell layer and larger cell size of P*_SlBRI1_*::SlBRI1 at 3 DPA but was surpassed by those of P*_SlBRI1_*::T825A after that (Figure 5). Third, autophosphorylation of BRI1 is important for early events in BR signaling. In this study, the autophosphorylation level of T825A was much higher than that of SlBRI1, while T825D showed a nearly undetected autophosphorylation level similar to K916E, which suggested a negative role of Thr-825 in SlBRI1 autophosphorylation and subsequent BR signal strength. This finding was in agreement with previously mentioned results; most phosphorylation sites of *Arabidopsis* BRI1, such as Thr-1039, Ser-1044, and Thr-1049, exhibited strong functions in autophosphorylation; thus, preventing phosphorylation of these sites usually resulted in attenuated BR signaling and disturbed plant growth [28]. Thr-1050 in tomato SlBRI1 acted as a negative regulator since T1050A showed enhanced autophosphorylation of SlBRI1 and BR signal strength and promoted yield [29]. Thus, T825A exhibited a greater recovery of BR signaling than SlBRI1, which partly led to the elevated cell development in the P*_SlBRI1_*::T825A lines.

Regulation of cell expansion is also mediated through crosstalk between BR signaling and other hormone signaling pathways, such as GA and IAA [8,49]. GA is a growth-promoting hormone that has overlapping functions with BR in multiple plant developmental processes and corresponding cellular activities [50,51]. In fruit developmental regulation, the application of GA could induce fruit with larger cells but fewer cell layers [52]. Stimulation of cell elongation by GA required BR signaling, while the GA-deficient dwarf phenotype could be suppressed by BZR1. Further studies reported that BR and GA function interdependently, which accounted for cell elongation through the BZR1-DELLA module [53,54]. DELLA is the central repressor in the GA signaling pathway and interacts with the GA receptor GID1 to increase affinity upon GA treatment [55]. In tomato, the expression level of *SlDELLA* is low during the phase of fruit cell expansion, and the *SlDELLA* mutant *procera* exhibited enhanced GA-response phenotypes and thus a larger pericarp cell size, such as a larger ovary at −3 DPA, 0 DPA, and 3 DPA [56]. Furthermore, there is a feedback mechanism in which the expression of the GA-biosynthetic genes *GA20ox1* and *GID1* were downregulated by *DELLA*-deficient conditions or GA application to maintain GA homeostasis in plants [57,58,59,60,61]. Consistent with the above findings, after 5 DPA, the ovaries of P*_SlBRI1_*::T825A exhibited lower transcription levels of *SlGID1* and *SlDELLA* than those of the P*_SlBRI1_*::SlBRI1 ovaries (Figure 6), which could explain the larger cell size and larger fruit of the P*_SlBRI1_*::T825A lines (Figure 4 and Figure 5). Therefore, SlBRI1 Thr-825 influenced GA signaling by regulating the accumulation of SlDELLA, which subsequently affected cell expansion and fruit size determination.

Previous studies showed that the yield per plant increased when the SlBRI1 phosphorylation sites Thr-825, Thr-1050, and Ser-1040 were dephosphorylated. To determine whether these sites functioned in the same way, GA signaling of the P*_SlBRI1_*::T825A, P*_SlBRI1_*::S1040A, P*_SlBRI1_*::T1050A, and P*_SlBRI1_*::SlBRI1 lines was compared. The results showed that GA signaling in the P*_SlBRI1_*::T825A lines was highest among the lines, which indicated that the regulatory mechanism of each SlBRI1 phosphorylation site on fruit development is different, and only Thr-825 affected fruit development through GA signaling (Figure 7).

Moreover, BR was demonstrated to coordinate with IAA to control cell expansion through the phosphorylation and inhibition of AUXIN RESPONSE FACTOR-2 (ARF2) by BIN2 (BRASSINOSTEROID INSENSITIVE 2) [62]. However, the expression of IAA-related genes, such as *SlIAA17* and *SlARF2A*, was unchanged among all the transgenic plants in this study (Figure S4). In addition, the transcription levels of the cell growth-regulated gene *SlEXPA5* (*α-expansin 5*), as well as *FW2.2* and *FW3.2*, which are considered to influence fruit weight by regulating cell division at the early fruit development stage, were not altered between the P*_SlBRI1_*::T825A and P*_SlBRI1_*::SlBRI1 lines (Figure S4) [63,64].

## 4. Materials and Methods

### 4.1. Amino Acid Sequence Alignment

Multiple amino acid sequence alignment of SlBRI1 homologs was performed using ClustalX2 (clustal.org/download, accessed on 24 December 2020). MEME software (http://meme-suite.org/tools/meme, accessed on 30 December 2020) was used to analyze the motifs. The parameters of the optimum motif width and the maximum number of motifs were 6 and 30 wide and 30, respectively, and the other parameters were the default values.

### 4.2. Site-Directed Mutagenesis and Vector Construction

For site-directed mutagenesis, full-length *SlBRI1* (*Solyc*04g051510) was amplified from tomato (*S. lycopersicum* cv. Moneymaker) and used as a template. T825A (in which Thr-825 was replaced with alanine), T825D (in which Thr-825 was replaced with aspartic acid), and K916E (in which Lys-916 was replaced with glutamic acid) were amplified from wild-type SlBRI1 using overlap PCR amplification.

For plant expression vector construction, full-length *SlBRI1* and T825A were recombined into the binary transformation vector pBI121 (Clontech, Palo Alto, CA, USA) with a GFP tag followed by its CT region. The native promoter of *SlBRI1* (−2989 bp) was also cloned into pBI121 to replace the 35S promoter. The recombined vectors were transformed into *Agrobacterium tumefaciens* strain GV3101 for subsequent tomato transformation. For prokaryotic expression vector construction, the cytoplasmic domains (824 aa to 1207 aa) of SlBRI1, T825, T825D, and K916E were recombined into the prokaryotic expression vector pFLAG-MAC (Sigma-Aldrich Saint Louis, MO, USA). The recombinant vectors were transformed into *Escherichia coli* BL21 (DE3) pLysS (Transgene, CD901-02, Beijing, China) for subsequent autophosphorylation analysis. All primers designed for site-directed mutagenesis and vector construction are listed in Table S1.

### 4.3. Tomato Transformation

The cotyledon transformation method was used for tomato transformation [65], while the weak SlBRI1 mutant *cu3^-abs1^* with a missense mutation in His-1012 was the transgenic acceptor. PCR analysis and quantitative real-time PCR were used to screen the transgenic plants, and two independent homozygous lines from both P*_SlBRI1_*::SlBRI1 (SlBRI1-1 and SlBRI1-3 hereafter) and P*_SlBRI1_*::T825A (T825A-1 and T825A-2 hereafter) were advanced to the T2 generation for further analyses in this study. Transgenic plants expressing P*_SlBRI1_*::T1050A (in which Thr-1050 of SlBRI1 was replaced with alanine) and P*_SlBRI1_*::S1040A (in which Ser-1040 of SlBRI1 was replaced with alanine) were both from previous reports [29,30].

### 4.4. Agronomic Trait Characterization

For germination assays, the seeds of both transgenic lines and *cu3^-abs1^* were inoculated in Petri dishes with wet filter paper at 28 °C in the dark as previously reported [30]. The germination rate and germination potential were calculated on the seventh day and third day after seeding, respectively. There were three replicates for each index, and each replicate had 100 seeds.

All the plants for agronomic trait investigation were grown in a greenhouse. Plant height and plant expansion were the distance from the top of the plant to the ground and the maximum diameter of the plant, respectively. The stem diameter and internodal length were the diameter and length of the third node from bottom to top per plant, respectively. All vegetative growth-related traits were measured at the mature stage (maturation period of the fourth fruit cluster of the P*_SlBRI1_*::T825A lines). Fruit yield was the total weight of the first to the fourth fruit nodes. Single fruits at the red ripening stage were harvested to measure fruit weight, fruit volume, and fruit shape index, and the fruit shape index was the ratio of the longitudinal diameter to the transverse diameter of the fruit.

The fruit number per cluster was the fruit number of the third cluster. Ovary diameters at 1 day post-anthesis (DPA), 3 DPA, 5 DPA, and 7 DPA were measured using ImageJ software (NIH, Bethesda, MD, USA). At least 6 plants for each line were used for fruit yield investigation, while investigations of vegetative growth-related traits and reproductive growth-related traits included at least 10 and 15 plants, respectively, for each line.

### 4.5. Paraffin Section Observation

At least 5 resin equatorial sections of the ovary walls for each developmental stage from the P*_SlBRI1_*::SlBRI1, P*_SlBRI1_*::T825A, and *cu3^-abs1^* ovaries were isolated to observe the cell size and the number of cell layers via paraffin sectioning. The samples were fixed with formalin-acetic acid fixative (70% ethyl alcohol: glacial acetic acid: formalin at a ratio of 90:5:5) and stained with hematoxylin (Sigma, H9627). The stained samples were then dehydrated in a graded series of ethyl alcohol (70%, 85%, 95%, and 100%), followed by clearing with a graded series of chloroform (chloroform: ethyl ethanol in a ratio of 1:3, 1:1, 3:1, and 100% chloroform). Paraffin was immersed in chloroform at 37 °C, and the samples were embedded. Ten-micrometer-thick sections were cut using a microtome (RM2265, Leica, Wetzlar, Germany), dewaxed with xylene, and observed using a microscope (M165 FC, Leica, Wetzlar, Germany). Cell size was quantified using ImageJ software, while the number of cell layers was counted manually.

### 4.6. Hypocotyl Elongation Response to Exogenous BL and BRZ

Seeds of P*_SlBRI1_*::SlBRI1, P*_SlBRI1_*::T825A, and *cu3^-abs1^* were sterilized and inoculated in Petri dishes containing solid 1/2 strength Murashige and Skoog medium (MS, PhytoTechnology Laboratories, M519, Lenexa, KS, USA) with 0 nM, 10 nM, 100 nM, 500 nM, 1000 nM, and 2000 nM exogenous 24-epibrassinolide (epi-BL, Shanghai Yuanye Biotechnology Co., Ltd., Shanghai, China) or BR inhibitor brassinazole (BRZ, Tokyo Chemical Industry Co., Ltd., Tokyo, Japan). These seeds were grown at 25 °C in the dark for 10 days, and the hypocotyl lengths were then measured. The relative hypocotyl length was the change in hypocotyl length at different concentrations of epi-BL or BRZ. At least 15 seedlings from each concentration group were examined.

### 4.7. Relative Expression Analysis

Total RNA from various tissues including the second leaves at the six-leaf stage, 1 DPA ovaries, 3 DPA ovaries, 5 DPA ovaries, and 7 DPA ovaries from both the transgenic and *cu3^-abs1^* plants were extracted and transcribed to cDNA as previously described [30]. For quantitative real-time PCR (qRT-PCR) analysis, the program was carried out using a SYBR Green Master Mix Kit (Vazyme, Q121-02, Nanjing, China) according to the manufacturer’s protocol. The relative expression level was calculated by using the 2^−^^△△CT^ method, and each data point had 3 biological and technical replications. For semiquantitative reverse transcription PCR (RT-PCR), PCR amplification was performed following the manufacturer’s protocol. The amplified products were analyzed by agarose gel electrophoresis, and each product had 3 biological replications. The tomato *SlACTIN* gene was used as an internal control for both qRT-PCR and RT-PCR, and all primer sequences were described previously and are listed in Table S1 [2,30,39,66].

### 4.8. Autophosphorylation Analysis In Vitro

The recombinant proteins from *E. coli* BL21 (DE3) pLysS cells with SlBRI1-pFLAG-MAC, T825A-pFLAG-MAC, T825D-pFLAG-MAC, or K916E-pFLAG-MAC were extracted and purified by anti-FLAG M2 affinity gels (A2220, Sigma-Aldrich, Saint Louis, MO, USA). Anti-FLAG antibodies (Transgene, Beijing, China) were subsequently used to detect the loading control of the recombinant proteins, followed by anti-pThr antibodies (#93815, CST, Danvers, MA, USA) to detect their autophosphorylation levels in vitro. SlBRI1-pFLAG-MAC and K916E-pFLAG-MAC were the positive and negative controls, respectively. The experiments were repeated 3 times and the detailed protocols were performed as previously described [28,67].

### 4.9. Statistical Analysis

Data analysis in this study was carried out using SigmaPlot 12.0 (Systat Software Inc., Chicago, IL, USA). Significant differences were analyzed by one-way analysis of variance (ANOVA) and Tukey’s test via DPS 7.05. Mean and standard error values were calculated, and the different letters indicate significant differences at *p* < 0.05.

## 5. Conclusions

Tomato fruit growth is a key biological process because of its close link with high yield. BRs are considered to have good agricultural potential since they can promote fruit growth by stimulating cell division and expansion. However, BR usually functions positively at nanomolar concentrations, and concentrations that are too high and too low result unstable effects. Thus, appropriate BR signal strength control is a key factor for the potential of BR in tomato breeding. BRI1 is the major BR receptor kinase, and its phosphorylation sites gradually influence BR signal strength and plant botanic characteristics. In this research, the agronomic potential and molecular mechanism of SlBRI1 Thr-825 in tomato were revealed. Mimic nonphosphorylation of Thr-825 could activate the autophosphorylation level of SlBRI1, thereby enhancing BR signaling and the subsequent expression level of *SlBZR1*. The expression of *SlDELLA* was then repressed accordingly and led to enhanced GA signal strength. Cell division and expansion were subsequently changed because of the comprehensive effect of these factors and eventually led to the larger plant shape and increased fruit yield of tomato. Higher GA signaling in mimic nonphosphorylation of SlBRI1 Thr-825 might contribute to a unique regulatory mechanism for increasing tomato yield than mimic nonphosphorylation of SlBRI1 Thr-1050 and Ser-1040. These results reveal the agronomic value of SlBRI1 Thr-825 in tomato breeding and provide a new candidate phosphorylation site of SlBRI1 that can increase crop yields by precisely fine-tuning BR signal strength.

## Data Availability

Not applicable.

## Supplementary Materials

The following are available online at https://www.mdpi.com/article/10.3390/ijms22147673/s1.

## Abbreviations

BR	Brassinosteroid
BRI1	Brassinosteroid Insensitive1
IAA	Indole-3-acetic acid
GA	Gibberellic acid
Thr	Threonine
Ser	Serine
Tyr	Tyrosine
CDK	Cyclin-dependent kinase
LRR-RLK	Leucine-rich repeat receptor-like kinase
BZR1	BRASSINAZOLERESISTANT1
BES1	BRI1-EMS SUPPRESSOR1
T825A	Threonine-825-alanine
T825D	Threonine-825-aspartic acid
K916E	Lysine-916-Glutamic acid
S1040A	Serine-1040- alanine
T1050A	Threonine-1050-alanine
GFP	Green fluorescent protein
epi-BL	24-Epibrassinolide
BRZ	Brassinazole
CPD	Constitutive Photomorphogenesis and Dwarf
DWARF	6-DEOXOCASTASTERONE OXIDASE
GID1	Gibberellin Insensitive Dwarf 1
ARF2	AUXIN RESPONSE FACTOR-2
BIN2	BRASSINOSTEROID INSENSITIVE 2
qRT-PCR	Quantitative real-time PCR analysis
MS	Murashige and Skoog medium

## References

[1] Azzi L., Deluche C., Gevaudant F., Frangne N., Delmas F., Hernould M., Chevalier C. (2015). Fruit growth-related genes in tomato. J. Exp. Bot..

[2] Zhang S., Xu M., Qiu Z., Wang K., Du Y., Gu L., Cui X. (2016). Spatiotemporal transcriptome provides insights into early fruit development of tomato (*Solanum lycopersicum*). Sci. Rep..

[3] Pattison R.J., Csukasi F., Zheng Y., Fei Z., van der Knaap E., Catala C. (2015). Comprehensive Tissue-Specific Transcriptome Analysis Reveals Distinct Regulatory Programs during Early Tomato Fruit Development. Plant Physiol..

[4] Joubes J., Phan T.H., Just D., Rothan C., Bergounioux C., Raymond P., Chevalier C. (1999). Molecular and biochemical characterization of the involvement of cyclin-dependent kinase A during the early development of tomato fruit. Plant Physiol..

[5] Dewitte W., Murray J.A.H. (2003). The plant cell cycle. Annu. Rev. Plant Biol..

[6] Enders T.A., Strader L.C. (2015). Auxin Activity: Past, Present, and Future. Am. J. Bot..

[7] Frick E.M., Strader L.C. (2018). Roles for IBA-derived auxin in plant development. J. Exp. Bot..

[8] Oh M.H., Honey S.H., Tax F.E. (2020). The Control of Cell Expansion, Cell Division, and Vascular Development by Brassinosteroids: A Historical Perspective. Int. J. Mol. Sci..

[9] Chen S., Wang X.J., Tan G.F., Zhou W.Q., Wang G.L. (2020). Gibberellin and the plant growth retardant Paclobutrazol altered fruit shape and ripening in tomato. Protoplasma.

[10] Li Z.C., He Y.H. (2020). Roles of Brassinosteroids in Plant Reproduction. Int. J. Mol. Sci..

[11] Liu L., Jia C., Zhang M., Chen D., Chen S., Guo R., Guo D., Wang Q. (2014). Ectopic expression of a BZR1-1D transcription factor in brassinosteroid signalling enhances carotenoid accumulation and fruit quality attributes in tomato. Plant Biotechnol. J..

[12] Nolan T., Chen J.N., Yin Y.H. (2017). Cross-talk of Brassinosteroid signaling in controlling growth and stress responses. Biochem. J..

[13] Zhiponova M.K., Vanhoutte I., Boudolf V., Betti C., Dhondt S., Coppens F., Mylle E., Maes S., Gonzalez-Garcia M.P., Cano-Delgado A.I. (2013). Brassinosteroid production and signaling differentially control cell division and expansion in the leaf. New Phytol..

[14] De Rybel B., Audenaert D., Vert G., Rozhon W., Mayerhofer J., Peelman F., Coutuer S., Denayer T., Jansen L., Nguyen L. (2009). Chemical Inhibition of a Subset of Arabidopsis thaliana GSK3-like Kinases Activates Brassinosteroid Signaling. Chem. Biol..

[15] Li J.M., Chory J. (1997). A putative leucine-rich repeat receptor kinase involved in brassinosteroid signal transduction. Cell.

[16] Kim T.W., Guan S.H., Sun Y., Deng Z.P., Tang W.Q., Shang J.X., Sun Y., Burlingame A.L., Wang Z.Y. (2009). Brassinosteroid signal transduction from cell-surface receptor kinases to nuclear transcription factors. Nat. Cell Biol..

[17] Clouse S.D. (2011). Brassinosteroid Signal Transduction: From Receptor Kinase Activation to Transcriptional Networks Regulating Plant Development. Plant Cell.

[18] Wang Z.Y., Wang Q., Chong K., Wang F., Wang L., Bai M., Jia C. (2006). The brassinosteroid signal transduction pathway. Cell Res..

[19] Yamamuro C., Ihara Y., Wu X., Noguchi T., Fujioka S., Takatsuto S., Ashikari M., Kitano H., Matsuoka M. (2000). Loss of function of a rice brassinosteroid insensitive1 homolog prevents internode elongation and bending of the lamina joint. Plant Cell.

[20] Nakamura A., Fujioka S., Sunohara H., Kamiya N., Hong Z., Inukai Y., Miura K., Takatsuto S., Yoshida S., Ueguchi-Tanaka M. (2006). The role of OsBRI1 and its homologous genes, OsBRL1 and OsBRL3, in rice. Plant Physiol..

[21] Kir G., Ye H.X., Nelissen H., Neelakandan A.K., Kusnandar A.S., Luo A.D., Inze D., Sylvester A.W., Yin Y.H., Becraft P.W. (2015). RNA Interference Knockdown of BRASSINOSTEROID INSENSITIVE1 in Maize Reveals Novel Functions for Brassinosteroid Signaling in Controlling Plant Architecture. Plant Physiol..

[22] Hou Q., Saima S., Ren H., Ali K., Bai C., Wu G., Li G. (2019). Less Conserved LRRs Is Important for BRI1 Folding. Front. Plant Sci..

[23] Oh M.H., Wang X.F., Kota U., Goshe M.B., Clouse S.D., Huber S.C. (2009). Tyrosine phosphorylation of the BRI1 receptor kinase emerges as a component of brassinosteroid signaling in Arabidopsis. Proc. Natl. Acad. Sci. USA.

[24] Oh M.H., Sun J.D., Oh D.H., Zielinski R.E., Clouse S.D., Huber S.C. (2011). Enhancing Arabidopsis Leaf Growth by Engineering the BRASSINOSTEROID INSENSITIVE1 Receptor Kinase. Plant Physiol..

[25] Wang Q.N., Wang S.F., Gan S.F., Wang X., Liu J.W., Wang X.F. (2016). Role of Specific Phosphorylation Sites of Arabidopsis Brassinosteroid-Insensitive 1 Receptor Kinase in Plant Growth and Development. J. Plant Growth Regul..

[26] Bajwa V.S., Wang X.F., Blackburn R.K., Goshe M.B., Mitra S.K., Williams E.L., Bishop G.J., Krasnyanski S., Allen G., Huber S.C. (2013). Identification and Functional Analysis of Tomato BRI1 and BAK1 Receptor Kinase Phosphorylation Sites. Plant Physiol..

[27] Nie S., Huang S., Wang S., Cheng D., Liu J., Lv S., Li Q., Wang X. (2017). Enhancing Brassinosteroid Signaling via Overexpression of Tomato (*Solanum lycopersicum*) SlBRI1 Improves Major Agronomic Traits. Front. plant Sci..

[28] Wang X.F., Goshe M.B., Soderblom E.J., Phinney B.S., Kuchar J.A., Li J., Asami T., Yoshida S., Huber S.C., Clouse S.D. (2005). Identification and functional analysis of in vivo phosphorylation sites of the Arabidopsis BRASSINOSTEROID-INSENSITIVE1 receptor kinase. Plant Cell.

[29] Wang S., Liu J., Zhao T., Du C., Nie S., Zhang Y., Lv S., Huang S., Wang X. (2019). Modification of Threonine-1050 of SlBRI1 regulates BR Signalling and increases fruit yield of tomato. BMC Plant Biol..

[30] Wang S., Hu T., Tian A., Luo B., Du C., Zhang S., Huang S., Zhang F., Wang X. (2020). Modification of Serine 1040 of SIBRI1 Increases Fruit Yield by Enhancing Tolerance to Heat Stress in Tomato. Int. J. Mol. Sci..

[31] Giovannoni J.J. (2004). Genetic regulation of fruit development and ripening. Plant Cell.

[32] Morinaka Y., Sakamoto T., Inukai Y., Agetsuma M., Kitano H., Ashikari M., Matsuoka M. (2006). Morphological alteration caused by brassinosteroid insensitivity increases the biomass and grain production of rice. Plant Physiol..

[33] Chono M., Honda I., Zeniya H., Yoneyama K., Saisho D., Takeda K., Takatsuto S., Hoshino T., Watanabe Y. (2003). A semidwarf phenotype of barley uzu results from a nucleotide substitution in the gene encoding a putative brassinosteroid receptor. Plant Physiol..

[34] Honda I., Zeniya H., Yoneyama K., Chono M., Kaneko S., Watanabe Y. (2003). *Uzu* mutation in barley (*Hordeum vulgare* L.) reduces the leaf unrolling response to brassinolide. Biosci. Biotech. Biochem..

[35] Gillaspy G., Bendavid H., Gruissem W. (1993). Fruits—A Developmental Perspective. Plant Cell.

[36] Jiang J.R., Clouse S.D. (2001). Expression of a plant gene with sequence similarity to animal TGF-beta receptor interacting protein is regulated by brassinosteroids and required for normal plant development. Plant J..

[37] Joubes J., Walsh D., Raymond P., Chevalier C. (2000). Molecular characterization of the expression of distinct classes of cyclins during the early development of tomato fruit. Planta.

[38] Baldet P., Hernould M., Laporte F., Mounet F., Just D., Mouras A., Chevalier C., Rothan C. (2006). The expression of cell proliferation-related genes in early developing flowers is affected by a fruit load reduction in tomato plants. J. Exp. Bot..

[39] Czerednik A., Busscher M., Bielen B.A.M., Wolters-Arts M., de Maagd R.A., Angenent G.C. (2012). Regulation of tomato fruit pericarp development by an interplay between CDKB and CDKA1 cell cycle genes. J. Exp. Bot..

[40] Andersen S.U., Buechel S., Zhao Z., Ljung K., Novak O., Busch W., Schuster C., Lohmann J.U. (2008). Requirement of B2-type cyclin-dependent kinases for meristem integrity in *Arabidopsis thaliana*. Plant Cell.

[41] Gruszka D. (2013). The Brassinosteroid Signaling Pathway-New Key Players and Interconnections with Other Signaling Networks Crucial for Plant Development and Stress Tolerance. Int. J. Mol. Sci..

[42] Baghel M., Nagaraja A., Srivastav M., Meena N.K., Kumar M.S., Kumar A., Sharma R.R. (2019). Pleiotropic influences of brassinosteroids on fruit crops: A review. Plant Growth Regul..

[43] Clouse S.D., Sasse J.M. (1998). Brassinosteroids: Essential regulators of plant growth and development. Annu. Rev. Plant Phys..

[44] Clouse S. (2001). Brassinosteroids. Curr. Biol..

[45] Wang F.R., Bai M.Y., Deng Z.P., Oses-Prieto J.A., Burlingame A.L., Lu T.G., Chong K., Wang Z.Y. (2010). Proteomic Study Identifies Proteins Involved in Brassinosteroid Regulation of Rice Growth. J. Integr. plant Biol..

[46] Montoya T., Nomura T., Farrar K., Kaneta T., Yokota T., Bishop G.J. (2002). Cloning the tomato curl3 gene highlights the putative dual role of the leucine-rich repeat receptor kinase tBRI1/SR160 in plant steroid hormone and peptide hormone signaling. Plant Cell.

[47] Mathur J., Molnar G., Fujioka S., Takatsuto S., Sakurai A., Yokota T., Adam G., Voigt B., Nagy F., Maas C. (1998). Transcription of the Arabidopsis CPD gene, encoding a steroidogenic cytochrome P450, is negatively controlled by brassinosteroids. Plant J..

[48] Youn J.H., Kim T.W., Joo S.H., Son S.H., Roh J., Kim S., Kim T.W., Kim S.K. (2018). Function and molecular regulation of DWARF1 as a C-24 reductase in brassinosteroid biosynthesis in Arabidopsis. J. Exp. Bot..

[49] Fenn M.A., Giovannoni J.J. (2021). Phytohormones in fruit development and maturation. Plant J..

[50] Depuydt S., Hardtke C.S. (2011). Hormone Signalling Crosstalk in Plant Growth Regulation. Curr. Biol..

[51] Tanaka K., Nakamura Y., Asami T., Yoshida S., Matsuo T., Okamoto S. (2003). Physiological roles of brassinosteroids in early growth of Arabidopsis: Brassinosteroids have a synergistic relationship with gibberellin as well as auxin in light-grown hypocotyl elongation. J. Plant Growth Regul..

[52] Hu J.H., Israeli A., Ori N., Sun T.P. (2018). The Interaction between DELLA and ARF/IAA Mediates Crosstalk between Gibberellin and Auxin Signaling to Control Fruit Initiation in Tomato. Plant Cell.

[53] De Lucas M., Daviere J.M., Rodriguez-Falcon M., Pontin M., Iglesias-Pedraz J.M., Lorrain S., Fankhauser C., Blazquez M.A., Titarenko E., Prat S. (2008). A molecular framework for light and gibberellin control of cell elongation. Nature.

[54] Bai M.Y., Shang J.X., Oh E., Fan M., Bai Y., Zentella R., Sun T.P., Wang Z.Y. (2012). Brassinosteroid, gibberellin and phytochrome impinge on a common transcription module in *Arabidopsis*. Nat. Cell Biol..

[55] Shinozaki Y., Ezura K., Hu J.H., Okabe Y., Benard C., Prodhomme D., Gibon Y., Sun T.P., Ezura H., Ariizumi T. (2018). Identification and functional study of a mild allele of SlDELLA gene conferring the potential for improved yield in tomato. Sci. Rep..

[56] Carrera E., Ruiz-Rivero O., Peres L.E.P., Atares A., Garcia-Martinez J.L. (2012). Characterization of the procera Tomato Mutant Shows Novel Functions of the SlDELLA Protein in the Control of Flower Morphology, Cell Division and Expansion, and the Auxin-Signaling Pathway during Fruit-Set and Development. Plant Physiol..

[57] Sun T.P., Gubler F. (2004). Molecular mechanism of gibberellin signaling in plants. Annu. Rev. Plant Biol..

[58] Griffiths J., Murase K., Rieu I., Zentella R., Zhang Z.L., Powers S.J., Gong F., Phillips A.L., Hedden P., Sun T.P. (2006). Genetic characterization and functional analysis of the GID1 gibberellin receptors in *Arabidopsis*. Plant Cell.

[59] Hirano K., Ueguchi-Tanaka M., Matsuoka M. (2008). GID1-mediated gibberellin signaling in plants. Trends Plant Sci..

[60] Li J., Yu C., Wu H., Luo Z., Ouyang B., Cui L., Zhang J., Ye Z. (2015). Knockdown of a JmjC domain-containing gene JMJ524 confers altered gibberellin responses by transcriptional regulation of GRAS protein lacking the DELLA domain genes in tomato. J. Exp. Bot..

[61] Li J., Sima W., Ouyang B., Wang T., Ziaf K., Luo Z., Liu L., Li H., Chen M., Huang Y. (2012). Tomato SlDREB gene restricts leaf expansion and internode elongation by downregulating key genes for gibberellin biosynthesis. J. Exp. Bot..

[62] Vert G., Walcher C.L., Chory J., Nemhauser J.L. (2008). Integration of auxin and brassinosteroid pathways by Auxin Response Factor 2. Proc. Natl. Acad. Sci. USA.

[63] Frary A., Nesbitt T.C., Frary A., Grandillo S., van der Knaap E., Cong B., Liu J.P., Meller J., Elber R., Alpert K.B. (2000). fw2.2: A quantitative trait locus key to the evolution of tomato fruit size. Science.

[64] Park C.H., Kim T.W., Son S.H., Hwang J.Y., Lee S.C., Chang S.C., Kim S.H., Kim S.W., Kim S.K. (2010). Brassinosteroids control AtEXPA5 gene expression in *Arabidopsis thaliana*. Phytochemistry.

[65] Park S.H., Morris J.L., Park J.E., Hirschi K.D., Smith R.H. (2003). Efficient and genotype-independent *Agrobacterium*-mediated tomato transformation. J. Plant Physiol..

[66] De Jong M., Wolters-Arts M., Garcia-Martinez J.L., Mariani C., Vriezen W.H. (2011). The Solanum lycopersicum AUXIN RESPONSE FACTOR 7 (SlARF7) mediates cross-talk between auxin and gibberellin signalling during tomato fruit set and development. J. Exp. Bot..

[67] Li J., Wen J.Q., Lease K.A., Doke J.T., Tax F.E., Walker J.C. (2002). BAK1, an Arabidopsis LRR receptor-like protein kinase, interacts with BRI1 and modulates brassinosteroid signaling. Cell.

