# Supplementary material for: Modification of Threonine-825 of SlBRI1 Enlarges Cell Size to Enhance Fruit Yield by Regulating the Cooperation of BR-GA Signaling in Tomato
Source: Int J Mol Sci. 2021 Jul 18;22(14):7673. doi: 10.3390/ijms22147673 (PMC8305552; doi:10.3390/ijms22147673)
Supplement: Supplementary file 1 [file ijms-22-07673-s001.zip › ijms-1290129-supplementary.pdf]

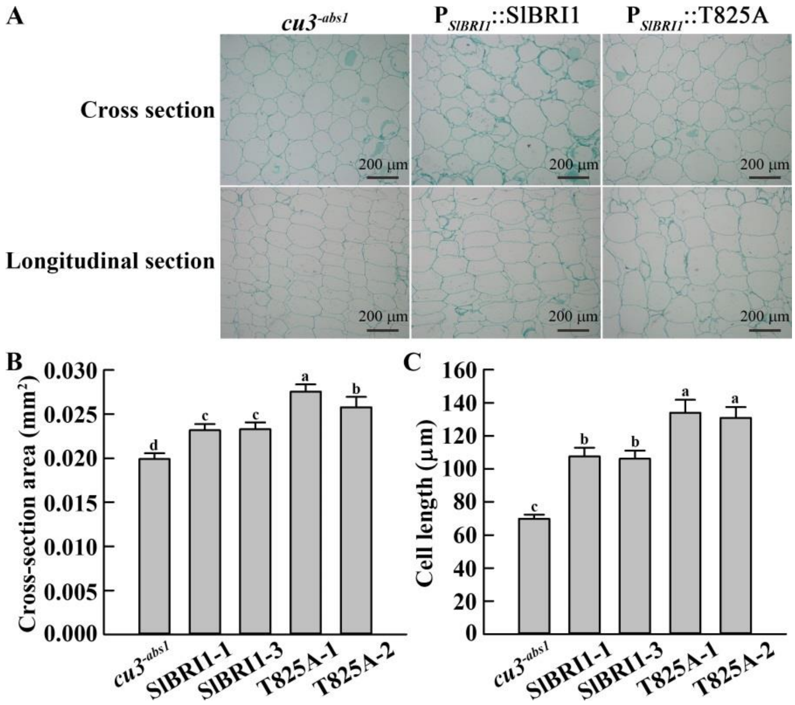

Figure S1. SlBRI1 T825A affected the cell size of the stems. (**A**) Cross section (top) and longitudinal section (bottom) of stems from both the transgenic and $cu3^{-abs1}$ plants. Scale bars represent 200 μm. (**B**) The cross-section area of the stem per plant. (**C**) The cell length of the stem per plant. The data for (**B**, **C**) are presented as the mean ± SD (n=5). The different letters indicate significant differences (one-way ANOVA, Tukey's test, p <0.05).

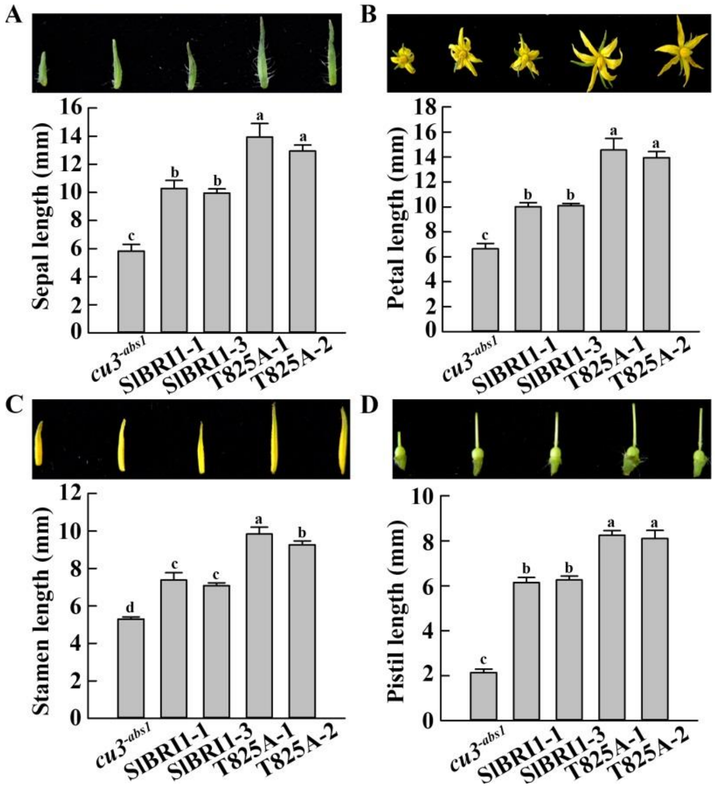

Figure S2. SlBRI1 T825A affected the development of the floral organs. (**A**) Top, phenotypes of a single

sepal per plant. Bottom, sepal length per plant. (**B**) Top, phenotypes of a single petal per plant. Bottom, petal length per plant. (**C**) Top, phenotypes of a single stamen per plant. Bottom, stamen length per plant. (**D**) Top, phenotypes of a single pistil per plant. Bottom, pistil length per plant. The floral organs of (**A**-**D**) shown on the top from left to right are as follows: *cu3*$^{-abs1}$, SlBRI1-1, SlBRI1-3, T825A-1, and T825A-2. The data for (**A**-**D**) are presented as the mean ± SD (n=6). The different letters indicate significant differences (one-way ANOVA, Tukey's test, p <0.05).

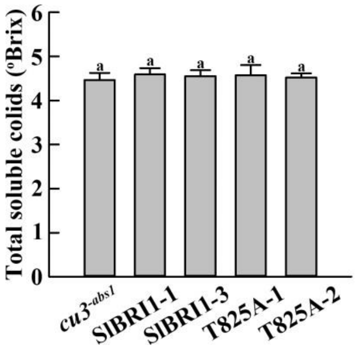

Figure S3. SlBRI1 T825A had no influence on fruit quality during the red ripe period. The data are presented as the mean ± SD (n=15). The different letters indicate significant differences (one-way ANOVA, Tukey's test, p <0.05).

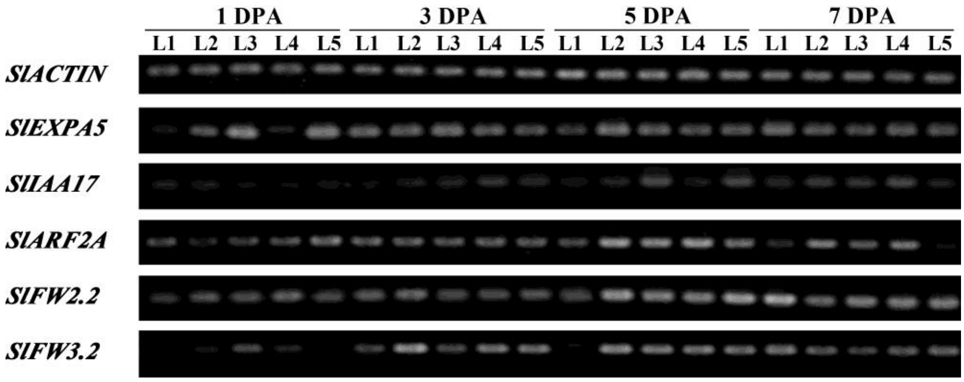

Figure S4. SlBRI1 T825A had no influence on the relative transcript levels of *SlIAA17*, *SlARF2A*, *SlEXPA5*, *FW2.2* and *FW3.2*. *SlACTIN* was used as a reference gene in tomato. Total RNA from 1 DPA, 3 DPA, 5 DPA, and 7 DPA ovaries from both the transgenic and *cu3*$^{-abs1}$ plants was extracted and transcribed to cDNA. The relative transcript levels were tested by RT-PCR. L1 (Line 1), *cu3*$^{-abs1}$; L2 (Line 2), SlBRI1-1; L3 (Line 3), SlBRI1-3; L4 (Line 4), T825A-1; L5 (Line 5), T825A-2. Each product had 3 biological replications.

Table S1. Primers sequence used in this research

Primer name	Primer sequence (5'→3')
Promoter of *SlBRI1*-F	GACCATGATTACGCCAAGCTTCT CCATTTCAATTATTGCTCAAAGG
Promoter of *SlBRI1*-R	ACCACCCGGGGATCCTCTAGACT TCAAAGATTGAAACTTTATAGCTTAAA
SlBRI1-F for tomato transformation	TCTTTGAAGTCTAGAGCTCGAGAT GAAAGCTCACAAAACTGTGTTTAAC
SlBRI1-R for tomato transformation	GCCCTTGCTCACCATGGTACCCTTA TCGTCGTCATCCTTGTAATC
T825A-F for site mutation	GTTGCCATAGAGGCGAAGAAGAGGA
T825A-R for site mutation	TCCTCTTCTTCGCCTCTATGGCAAC
T825D-F for site mutation	GTTGCCATAGAGGACAAGAAGAGGA
T825D-R for site mutation	TCCTCTTCTTGTCCTCTATGGCAAC
K916E-F for site mutation	GAGTGTTGTAGCTATTGAGAAATTGATAC ACG
K916E-R for site mutation	CGTGTATCAATTTCTCAATAGCTACAACA CTC
SlBRI1-CD-F for phosphorylation analysis in vitro	GACGATGACAAAGTCAAGCTTGAGA CGAAGAAGAGGAGGAGG
SlBRI1-CD-R for phosphorylation analysis in vitro	TCTGCAGGTACCCGGGAATTCAAGG TGTTTGCTCAGCTCATTG
SlACTIN-F for qRT-PCR and RT-PCR	GTGTGGGCTCACCTACGTTT
SlACTIN-R for qRT-PCR and RT-PCR	ACAATCCCAAGGGTTGTCAC
SlCPD-F for qRT-PCR and RT-PCR	ATCCAATTAACGTCCAACAT
SlCPD-R for qRT-PCR and RT-PCR	ACCTTTCATACACCTCCCTC
SlBRI1-F for qRT-PCR	TTCAATGGCACGATCCCGAA
SlBRI1-R for qRT-PCR	TGGGGAGAGGATACCCACAG
SlDWARF-F for qRT-PCR	ATTCAATGCTTTGATTGGCA
SlDWARF-R for qRT-PCR	GGGAGAGAAGTTGCAGGAAG
SlCDKB1-F for RT-PCR	ATGGAGAAATACGAGAAATTGGAG
SlCDKB1-R for RT-PCR	ACGATGTAGAGAGAATGAGATAGC
SlCDKB2.1-F for RT-PCR	ATGCTGGTAAGAGTGTATCGG
SlCDKB2.1-R for RT-PCR	CGGAGAGTAGTTGGAGGAAC
SlGA20ox1-F for RT-PCR	CTCATTTCTAATGCTCATCGT
SlGA20ox1-R for RT-PCR	R: TGCAGATGATTCTTTCTTAGCG
SlGID1-F for RT-PCR	GATCTTGATACACCTCTCAGTACTA
SlGID1-R for RT-PCR	ACAGCCTTACATATACTAACAAGAC
SlDELLA-F for RT-PCR	TGATGCGACTATACTTGATATAAG
SlDELLA-R for RT-PCR	GGGTTAATCTGTTTAATAGAGTTC
SlBZR1-F for RT-PCR	AAGTATGCGTCAAATGTTC
SlBZR1-R for RT-PCR	TGCTCTTACCCTTGTCTG
SlEXPA5-F for RT-PCR	AAGGGTTCAAGAACTCAATGGCAAC

SlEXPA5-R for RT-PCR	ACCATCGCCTGTAGTGACCTTAAAG
SlIAA17-F for RT-PCR	TTAAGGCTAGGGTTGCCTGG
SlIAA17-R for RT-PCR	AGCTGGGGTTTTGTGTGTTG
SlARF2A-F for RT-PCR	GGATGGCAAAGGGCATAGTA
SlARF2A-R for RT-PCR	TACGCTGCACCTCCTCTTTT
SlFW2.2-F for RT-PCR	CTGGGATTGACAGGATTGCCT
SlFW2.2-R for RT-PCR	TAGCTTGCCACCCTATTCCC
SlFW2.3-F for RT-PCR	ATTACTCTCGTGGGCAAGGC
SlFW2.3-R for RT-PCR	CTGAACCGAATGGTGCAAGC
